# Hospital and Institutional News

**Published:** 1919-06-07

**Authors:** 


### HOSPITAL AND INSTITUTIONAL NEWS.

### STATE CONTROL AND THE PATIENTS' PARAMOUNT INTERESTS.

Our readers have had sufficient time to study and consider the address by Sir Napier Burnett on "The Relations of Voluntary Hospitals to the State," which we published in our issue of May 24. In that paper an attempt was made to consider the hospital situation as a whole throughout the country, not merely metropolitan hospitals, or special hospitals, but all hospitals of every type, each group of which, and many individual hospitals, have their own conditions and problems. Sir Napier Burnett expressed a wish to take a broad outlook towards what is in the best interests of the nation, rather than the point of view of any one individual hospital. That represents one side of the serious question and problems involved. But there is another and an equally, if not more, important side, for it involves the best interests and the protection of hospital patients as a whole, and as individuals. It is the aim and glory of every well-managed voluntary hospital to put the patient first, and his or her best interests are made the first consideration in every properly administered voluntary hospital throughout the country. The paramount importance of maintaining this first principle in the administration of every hospital of whatever type throughout the United Kingdom has been forcibly and painfully illustrated by the many resulting injustices and cruelties which members of the public have experienced since the setting up of a system of National Health Insurance, and the establishment of what is known as the panel system of treatment. The voluntary system, as we have it in the United Kingdom to-day, so far as the patients are concerned, is regarded with wonderment and envy in many countries of the world, whose inhabitants are wholly or mainly dependent on a system of State-controlled and administered hospitals.

### THE LEAGUE OF MERCY.

His Royal Highness The Prince of Wales, with the consent of His Majesty the King, has accepted the position of Grand President of The League of Mercy, which Office was formerly held by King Edward VII and his present Majesty, and, during His Royal Highness's minority until the present time by the Earl of Athlone.

### COLONEL ADAMI AS VICE-CHANCELLOR.

Colonel John George Adami, M.A., M.D., D.Sc., LL.D., F.R.S., has been appointed Vice-Chancellor of Liverpool University in succession to Sir Albert Dale, who retires at the end of September. Colonel Adami, who was born in 1862, is a pathologist of the highest distinction. Since 1892 he has held the chair of pathology and bacteriology in the McGill University, Montreal. Colonel Adami has served in the Canadian Army Medical Corps as Assistant-Director of Medical Services, and is medical recorder for the Canadian Expeditionary Force.

### SUCCESSOR TO SIR GEORGE THANE.

Professor Sir George Thane, who has held the Chair of Anatomy at University College, London, since 1877, retires at the end of the current session. The Senate have expressed to him their deep sense of the benefits that Anatomical science has received from him. The Senate have appointed as his successor Professor Grafton Elliot Smith, who will take up his duties at University College in October next. Professor Elliot Smith graduated at Sydney, taking the M.D., with first-class honours and university medal, and at Cambridge, where he was a Fellow of St. John's College. He was formerly Professor of Anatomy in the Egyptian Government School of Medicine at Cairo, and since 1909 he has been Professor of Anatomy in the University of Manchester. He was awarded the Prix Fauvelle by the Société d'Anthropologie de Paris in 1911. He is a Fellow of the Royal Society, by whom he was awarded the Royal Medal in 1912. He is also a Fellow of the Royal College of Physicians, a member of the General Medical Council, and of several learned foreign societies. He has published a large number of memoirs on Anatomical and on Anthropological subjects.

### A CHAIR OF BACTERIOLOGY.

At a meeting of the College Board of the London Hospital on Monday, May 26, a letter was read from the Clerk to the Court of the Goldsmiths' Company offering, in the name of the Company, a sum of £15,000 for the endowment of a Chair of Bacteriology, to be known as the Goldsmiths' Company's Chair of Bacteriology. In making this gift the Court expressed the hope that their action might induce other organisations and individuals to consider the desirability of assisting the cause of medical education and research in a similar manner, a hope which all who have the interests of their country at heart must, we think, devoutly share, for in any attempt to remedy social evils, to prevent disease, or to improve the national physique, the existence and co-operation of a skilled and highly-trained medical personnel is obviously of primary importance. Such a personnel can only come from schools adequately staffed and equipped—in other words, adequately endowed. How deficient the London medical schools are in this last respect will be gathered from the fact that the present is, we believe, the first instance of the endowment of a Chair in connection with any subject of the medical curriculum at any one of them, and yet these are the schools which are probably the most favourably situated for the great purpose which they serve, for it is in London where, from the density of the population, disease is most abundant; it is in London where teachers and investigators most congregate, where the large medical museums and libraries are placed, and where scientific societies are most numerous and active; it is, in fact, in London where, granted those monetary sinews, as important in peace as in war, the medical educational centre of the world should be found. Nothing is, we think, better calculated to lead to the realisation of this prospect than gifts such as that which the Goldsmiths' Company have so munificently made to the London Hospital.

### SURPLUS ARMY MEDICAL STORES.

A catalogue has just been issued by the Surplus Government Property Disposal Board which contains much that is likely to be of interest and value to hospital managers. Land, buildings, furniture, iron, and so forth are only in exceptional cases likely to appeal to institutional buyers; but the medical stores section is one that should be carefully studied by every hospital secretary. The price of new instruments and appliances in this section is fixed at 10 per cent. under the prices laid down in the "Priced List of Medical Stores, 1917"; prices, it may be added, which are likely in many instances to be a good deal below those current at the present time. For used instruments and appliances the sale price is $33\frac{1}{3}$ per cent. below the official list already mentioned. It is thus clear that there are opportunities for buyers, and the sooner hospital managers make up their minds what they want, the more likely they are to get some of these Army surplus stores at the substantial reductions indicated.

### TEACHING OF OPHTHALMOLOGY.

In a report on the teaching and examination of medical students in diseases of the eyes the Council of British Ophthalmologists expresses the opinion that, at the time of qualification, the knowledge in these subjects possessed by the majorty of young British medical men is inadequate to enable them to diagnose and efficiently treat the cases met with in general practice. In common with many other "side branches" in medical work, this one has undoubtedly suffered from neglect, but the weakness lies in practical rather than theoretical causes. The opportunity exists in connection with almost every university and medical school for gaining the essential experiences in eye work, but there is—on the part of many examining bodies—no effort made to ensure that this opportunity be taken. There is not sufficient examination stimulus, and every teacher knows the small attention which is liable to be given under these conditions. The Council wisely advocate that a definite test in ophthalmology should be made an integral part of the qualifying examination by all those bodies which have not hitherto included it in their schedule. In view of the proposed new era of team work in medicine, a parallel recommendation might well be made in the instance of many other special sections of our hospitals, even at the expense of the earlier phases of the medical curriculum, if the whole cannot be lengthened beyond five years.

### SCIENCE AND THE ARMY.

Speaking at the recent annual dinner of the old students of the Royal College of Science, Sir Alfred Keogh paid warm tribute to the magnificent way in which workers in both medicine and pure science had combined to maintain the health of armies under every conceivable condition of strain and hardship. For science as linked to the organisation of the medical service they had had to rely on men outside the Army, and he was glad that realisation that the public services must rely upon the universities and great schools of the country for their research had led him, at the beginning of the war, to associate himself not only with what was best in so-called medical science, but in science in the most general sense. The fact that our armies, particularly under the stationary conditions of trench warfare, were not wiped out by disease was due entirely to scientific men—not only doctors, but engineers, chemists, and a host of others. The results of this wonderful co-ordination are by now universally appreciated, but we also realise that never before has been witnessed the extraordinary phenomenon of the British Army listening to such outside advice. Sir Alfred Keogh provided lucid explanations in his speech, but, whatever the true origin of such happenings, the fact remains that we see every sign of awakening and continued success along such co-ordinated lines, and feel that the originators, from the highest to the lowest, deserve the highest appreciation that the country can show.

### A "PHYSICIAN'S" ADVERTISEMENT.

The attention of the Editor of the *Times* is urgently required to an advertisement which has been allowed to appear on page 2 of the issue of that newspaper for June 4. This surprising advertisement runs as follows: "Cancer of 1st or 2nd Stage.—Vacancy in London Physician's house for case seeking cure without operation. Terms suitable to latter's income.—Write full description Box C 507, The Times Office, London, E.C. 4." We have not the slightest idea who the "London Physician" is, or what the nature of his treatment or of the accommodation he provides for resident patients. We make, therefore, no imputation of dishonesty against him; but we do say that he is at the least misguided, and that his advertisement is very seriously against the public interest. We say, further, that it is an advertisement which the managers of the *Times* should not have accepted; and one which, now their attention is drawn to it, we hope they will refuse in future. And if the London Physician is on the register of medical practitioners, we think the General Medical Council might very well inquire further into the matter.

### CONTAMINATED FOODS.

At an inquest held by the City Coroner at the Southwark Court on May 27, concerning the death of George Ward, a workman and formerly a soldier, the jury brought in a verdict of death due to syncope and shock caused by profound toxæmia following upon the ingestion of the contents of two boxes of tinned sardines. To this the jury added a unanimous rider suggesting the compulsory notification of all suspicious cases of food poisoning by the medical man in attendance, or even by the patient himself to the Medical Officer of Health. They requested the Coroner to forward that rider to (a) the President of the Local Government Board and (b) the L.C.C. Evidence showed that, following ingestion of the sardines, the deceased was seized with vomiting and severe colic, followed by diarrhœa, collapse and death. As so frequently happens in these instances, it had been found impossible to trace back the source of infection. The widow said she had destroyed the remains of the infected sardines, and the particular brand or house of purchase was unknown save to the deceased. The post-mortem examination made by the doctor prior to information of such death being given to the Coroner disclosed, however, marked inflammation and suffusion of blood in the small intestine. Dr. Waldo said it was of paramount importance in such cases for the doctor to notify the Medical Officer of Health, in view of early bacteriological examination of food remains being made and stoppage of further sales. We agree most heartily with the Coroner in the urgent need for such procedure, which, as we know, he has himself suggested many times during the past nineteen years. It is a point well worthy of attention in the coming health legislation.

### DEATH OF DR. WYNDHAM COTTLE.

THE death has occurred under rather tragic circumstances of Dr. Wyndham Cottle at Ningwood Manor, Isle of Wight. While Dr. Cottle, who took great interest in the welfare of animals, was in his garden, two favourite dogs began to fight. In attempting to separate them he was seized with heart failure and died. Formerly specialist on skin diseases at St. George's Hospital, London, Dr. Cottle has written several works, which are well known, on that subject. He was surgeon to the Scots Guards.

### THE INTERPRETATION OF "FELONY."

FROM a report by the Legal Assessor of the General Medical Council presented to that body at its recent sessions, it appears that by English law a prisoner charged with murder (presumably the same holds good for other crimes, too) and found by the jury to be "guilty, but insane," has not technically been convicted of felony. The extraordinary consequence follows that such an individual cannot be charged before the General Medical Council of any offence in a professional respect of which cognisance can be taken. The point arose over a special and very painful case, in which a practitioner was charged with murder, and a verdict as above was rendered. The result of the Legal Assessor's advice to the Council means that the name of this practitioner, who has been pronounced by a jury of his fellow-citizens to be not merely a murderer but insane into the bargain, cannot be removed from the medical register. For the present, doubtless, the public is protected against possible injury by his incarceration; but in the event of the prison authorities some day deciding that he is no longer insane, apparently he would be perfectly free to resume practice with the full status of a registered medical man.

### THE PRODUCTION OF ARMY CASE SHEETS.

A POINT which may not improbably govern a number of future cases arose before Mr. Justice Coleridge on May 30. The special matter referred to was the refusal of the War Office authorities to allow the production of certain medical history sheets concerning the health of the defendant in the case, who had been under treatment in a military hospital whilst serving in the Army. The Judge accepted this refusal as final, although he remarked on the service which it would have been to the Court to have had the sheets produced. Both parties to the suit, it should be added, desired the production of these sheets, so that no question of the revelation of professional secrets arose; and the War Office was quite willing that the individual medical officers concerned with the defendant's treatment should be subpœnaed to give such evidence as they could remember about their patient, though the refreshing of their memory by a perusal of their own notes in the official history sheets would be objected to. It is noteworthy that the assistance of a medical assessor was not obtained by the Court: it is difficult to understand the reluctance of judges to take advantage of a neutral expert opinion in difficult cases involving highly technical medical issues.

### QUEEN ALEXANDRA VISITS SEAMEN'S HOSPITAL.

ON Thursday, June 5, Queen Alexandra visited the Seamen's Hospital, Greenwich, and presented to the hospital's president, the Marquess of Milford Haven, a cheque for £10,000 to endow a ward as a memorial to seamen who lost their lives in the war. The sum was raised by the "Silver Thimble" Fund, and is one of the signs that the nation is beginning to realise how much it owed to the seamen during the war. History will probably record that the main cause of the Allied success in the struggle was the power of the blockade, and that could not have been made effective without the vigilance and heroism of the Navy and the men of the Mercantile Marine, who supported the Army, fed the nation and its allies, and strangled the Germans in a grip as deadly as it was silent. In view of this immense service, the Seamen's Hospital should never have to complain of want of support, but truth to tell the nation does not yet realise the magnificence of the feat accomplished by its fleets.

### THE MINISTRY OF HEALTH AND THE HOSPITALS.

ALTHOUGH at the time it was made we ridiculed the notion that the new Minister of Health would take over the voluntary hospitals, misgiving has already been expressed, and in some districts it has become difficult to persuade the public to continue its support, especially in regard to War Memorial schemes. Dr. Deas, of the Nelson Hospital, Wimbledon, has approached Mr. Hood, M.P., who has received the following reply from Dr. Addison: "I know of no intention," Dr. Addison wrote, "on the part of the Ministry of Health in the near future to take over the voluntary hospitals. . . .

I am not in a position to say whether the Ministry of Health will assist voluntary hospitals, or in what way." As we have said before, the moral is to make each hospital as complete and independent as possible before the Ministry of Health gets into its stride, so that each voluntary hospital may be as free as possible, not merely from departmental assistance, but from the conditions and inquisitions which that department might otherwise attempt to impose.

### THE MIGRATION OF CLIVEDEN HOSPITAL.

THE Public Health Committee of the Birmingham City Council has accepted, after a visit to Cliveden, the offer of Major Astor, M.P., of the temporary hospital and its equipment built on his estate at Taplow during the war, on condition that the hospital is transported to Birmingham. The institution was well known during the war when some 250 Canadians were accommodated there. After a visit which much impressed the representatives of the Committee, Major Astor's offer has been accepted, and should do something to reduce the heavy waiting list of which the Birmingham hospitals, not to mention the patients, have to complain. The offer is interesting as an example that public authorities on occasion do receive benefactions. We hope only that the new hospital when transported will form part of the scheme of co-ordination on which the Lord Mayor of Birmingham is now engaged.

### IMPROVEMENTS AT WEST HANTS HOSPITAL.

MR. CHILD CLARK has offered to build a nurses' Home for the Boscombe branch of the Royal Victoria and West Hants Hospital at a cost of £10,000. Needless to say this generous offer has been accepted. The salaries of the nursing staff have been raised already, and it is hoped to reduce their hours of work. It has also been decided to build a new ward at the Victoria branch in memory of the late Dr. Roberts Thomson. Plans have been drawn, and the public meeting which has approved the scheme is hoping to raise the necessary money. Further beds are badly needed, and discharged service men, under the Pensions Committee, are expected to occupy a large number.

### PERSISTENT DIFFICULTIES AT ENFIELD

A SOMEWHAT inconclusive meeting has been held at Enfield in connection with the Cottage Hospital, where, as we have already reported, the medical staff is at loggerheads with the Council, and has adopted a procedure in regard to the admission of cases which is, in effect, a defiance of the rules. By a majority of one, the report (which recorded the long average stay of the patients, namely twenty-nine days) was referred back to the Council. The former proposal of the medical staff for a conference between its representatives and the Council to revise certain rules was again proposed and rejected on the ground that, until the rules were obeyed by the staff, the Council could not meet the staff to discuss any revision. A resolution was also passed to empower the Council to appoint a general medical officer upon such terms and for such purpose as it may determine. On the whole the meeting supported Mr. F. W. Fletcher, the late chairman of the Council, and his colleagues; and if the medical staff does not fall into line, public support will inevitably be withdrawn, and the hospital will permanently suffer in reputation.

### CHESTER ROYAL INFIRMARY.

MR. J. ROWSE MITCHELL has been appointed Secretary to the Chester Royal Infirmary in succession to Mr. Wm. Rutter, who takes up his appointment at the Liverpool Royal Infirmary at the end of this month. Mr. Mitchell has been on the clerical staff of the South Devon and East Cornwall Hospital, Plymouth, since 1903. He enlisted in a field ambulance on the outbreak of war, and served continuously with that unit in France from November 1914 until May 1918, when he was taken prisoner by the enemy, being repatriated in January of the present year.

### OBSERVATION BEDS FOR TUBERCULOSIS.

THE London County Council has prepared a scheme for the provision at hospitals of beds for observation purposes in connection with the treatment of tuberculosis. As the Public Health Committee points out, it is very desirable to arrange for the establishment at hospitals of units of observation beds, both for the purpose of determining the suitability of cases for various forms of treatment, and for giving dispensary medical officers and general practitioners, at present deprived of the advantages arising from the observation of patients in residential institutions, opportunities of consulting with the hospital physician. The use of the hospitals in the manner suggested would, moreover, secure the services of the highly expert staffs of the hospitals and the advantages to be obtained from the co-ordinated services of the ear, nose and throat departments, the *x*-ray departments, and other special branches of investigation available at the hospitals. The idea is to get 100 beds at a cost of about £9,000 a year.

### THIS WEEK'S DRUG MARKET.

THE tone of the market is healthy and business is steadily improving. The result of the increased demand and the considerable number of inquiries from the Continent and from overseas for drugs has not only been to keep the trade on the downward movement of prices, but to send up prices in some cases. In some instances, however, prices continue to move downwards. Aspirin, antifebrin, salicylic acid, barbitone, and antipyrin, for instance, tend towards lower values. Following on the considerable drop in the value of Turkey opium, the prices of morphine and its salts tend downwards. Atropine is cheaper. Cocaine is more freely available, and the price is rather lower. The value of lithia salts is not so firmly maintained. Benzoates are cheaper. On the other hand, the value of menthol is firmly maintained. Calomel, corrosive sublimate, white precipitate and other salts of mercury are dearer. Linseed, linseed oil and castor oil continue to move up in price. Camphor is again dearer.

